# d-amino acid oxidase activator gene (*DAOA*) variation affects cerebrospinal fluid homovanillic acid concentrations in healthy Caucasians

**DOI:** 10.1007/s00406-012-0313-z

**Published:** 2012-03-28

**Authors:** Dimitrios Andreou, Peter Saetre, Thomas Werge, Ole A. Andreassen, Ingrid Agartz, Göran C. Sedvall, Håkan Hall, Lars Terenius, Erik G. Jönsson

**Affiliations:** 1 Department of Clinical Neuroscience, HUBIN Project, Karolinska Institutet and Hospital, R5:00, 171 76 Stockholm, Sweden; 2 Research Institute of Biological Psychiatry, Mental Health Center Sct. Hans, Copenhagen University Hospital, Roskilde, Denmark; 3 TOP Project, Division of Psychiatry, Ullevål University Hospital, University of Oslo, Oslo, Norway; 4 TOP Project, Institute of Clinical Medicine, Psychiatry Section Vinderen, University of Oslo, Oslo, Norway; 5 Department of Psychiatry, Diakonhjemmet Hospital, Oslo, Norway; 6 Institute of Psychiatry, University of Oslo, Oslo, Norway; 7 Department of Public Health and Caring Sciences, Uppsala University, Uppsala, Sweden

**Keywords:** d-amino acid oxidase activator gene (*DAOA*), Cerebrospinal fluid (CSF), Homovanillic acid (HVA), 5-hydroxyindoleacetic acid (5-HIAA), 3-methoxy-4-hydroxyphenylglycol (MHPG)

## Abstract

The d-amino acid oxidase activator (DAOA) protein regulates the function of d-amino oxidase (DAO), an enzyme that catalyzes the oxidative deamination of d-3,4-dihydroxyphenylalanine (D-DOPA) and d-serine. D-DOPA is converted to l-3,4-DOPA, a precursor of dopamine, whereas d-serine participates in glutamatergic transmission. We hypothesized that *DAOA* polymorphisms are associated with dopamine, serotonin and noradrenaline turnover in the human brain. Four single-nucleotide polymorphisms, previously reported to be associated with schizophrenia, were genotyped. Cerebrospinal fluid (CSF) samples were drawn by lumbar puncture, and the concentrations of the major dopamine metabolite homovanillic acid (HVA), the major serotonin metabolite 5-hydroxyindoleacetic acid (5-HIAA) and the major noradrenaline metabolite 3-methoxy-4-hydroxyphenylglycol (MHPG) were measured. Two of the investigated polymorphisms, rs3918342 and rs1421292, were significantly associated with CSF HVA concentrations. Rs3918342 was found to be nominally associated with CSF 5-HIAA concentrations. None of the polymorphisms were significantly associated with MHPG concentrations. Our results indicate that *DAOA* gene variation affects dopamine turnover in healthy individuals, suggesting that disturbed dopamine turnover is a possible mechanism behind the observed associations between genetic variation in *DAOA* and behavioral phenotypes in humans.

## Introduction

The *DAOA* (d-amino acid oxidase activator gene) is located on chromosome 13q34 and spans 29 Kb. This region, spanning 5 Mb, was initially investigated by Chumakov and colleagues, and two overlapping genes, *DAOA* (or *G72*) and *G30*, transcribed in opposing directions, were identified [7]. *DAOA* gene variation was initially associated with schizophrenia [7], and during the past decade, this association has been replicated in many subsequent studies (http://www.szgene.org) [2]. *DAOA* has also been associated with schizophrenia-related characteristics such as frontal lobe volume change [16], susceptibility to methamphetamine psychosis [26], response to antipsychotic treatment [36] and progression of prodromal syndromes to first episode psychosis [31]. Furthermore, *DAOA* has been associated with other psychiatric disorders and phenotypes such as major depression [40], bipolar disorder [38] and bipolar disorder severity [8]. An animal study, using *DAOA* transgenic mice, showed behavioral phenotypes associated with psychosis, some of which could be reversed with haloperidol [34].

The DAOA protein contains 153 amino acids and has been detected in various parts of the central nervous system (CNS), including amygdala, nucleus caudatus and spinal cord [7]. DAOA has also been implicated in the regulation of mitochondrial function and dendritic branching [28]. The DAOA protein was initially reported to behave as an activator of porcine d-amino acid oxidase (DAO), whereas more recent studies showed that DAOA modulates human DAO function as a negative effector [7, 42].

**Fig. 1:**
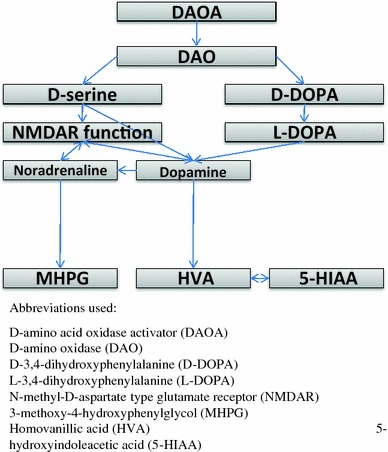
Biochemical and functional connections between d-amino acid oxidase activator and cerebrospinal fluid monoamine metabolites

DAO catalyzes the oxidative deamination of d-amino acids, such as d-3,4-dihydroxyphenylalanine (D-DOPA) and d-serine to α-keto acids. Thus, DAO deaminates D-DOPA to its corresponding α-keto acid, which is then transaminated to L-DOPA [24, 52]. L-DOPA then enters the basic biosynthetic pathway to dopamine and homovanillic acid (HVA). Dopamine is converted to noradrenaline by dopamine-beta-hydroxylase, and noradrenaline enters its basic catabolic pathway and is degraded to 3-methoxy-4-hydroxyphenylglycol (MHPG). Kinetic data show that the maximal velocity for the oxidative deamination of D-DOPA is much higher than for d-serine [24].

d-serine is an allosteric modulator of the *N*-methyl-d-aspartate (NMDA)-type glutamate receptors (NMDAR) [32], which have a modulatory site for d-serine. The occupation of this site by d-serine is required for glutamate to stimulate cation flow [19, 32]. Interaction between glutamate and noradrenaline [9] suggests that DAOA may be associated with noradrenaline via glutamatergic mechanisms (Fig. 1).

There is also evidence of a bidirectional interaction between NMDAR and the dopamine system. NMDAR activation leads to enhanced recruitment of the dopamine D1 receptor (DRD1) to the plasma membrane [37, 44]. Moreover, there is a direct protein–protein coupling between DRD1 and NMDAR [10, 29, 37]. It has been proposed that DRD1 and NMDAR early after their biosynthesis form heteromeric complexes, which are then transported to plasma membrane as preformed units [30]. NMDA antagonists lead to an increase in midbrain dopamine neuron firing rates [11], whereas striatal dopamine release has been reported increased or decreased in some studies [1, 39, 49]. A direct association between d-serine and dopamine release has also been shown, as high doses of d-serine attenuated amphetamine-induced dopamine release [46].

Taken together, there are biochemical connections between DAOA and the catecholamines dopamine and noradrenaline, via two identified pathways, first via DAO, d-serine and NMDAR, and secondly via DAO and D-DOPA (Fig. 1). The concentration of the major serotonin metabolite 5-hydroxyindoleacetic acid (5-HIAA) in cerebrospinal fluid (CSF) is strongly correlated with the major dopamine metabolite HVA [13]. This suggests that DAOA may also be associated with 5-HIAA (Fig. 1).

Given these biochemical and functional connections between the DAOA protein and the monoamine metabolites and the fact that studies in human twins and other primates indicate that monoamine metabolite concentrations are partly under genetic influence [17, 18, 35, 41], we can speculate that the well-established associations between *DAOA* and psychiatric phenotypes, described in the first paragraph, may be mediated by disturbed monoamine turnover rates.

In the present study, we aim to investigate whether *DAOA* polymorphisms are associated with dopamine, serotonin and noradrenaline turnover in the human brain. The concentrations of the major dopamine metabolite HVA, the major serotonin metabolite 5-HIAA and the major noradrenaline metabolite MHPG in CSF were used as indirect indexes of the monoamine turnover.

## Methods

### Subjects

Unrelated healthy Caucasians, 78 men and 54 women, participated in a longitudinal study. At the first interview, when CSF was sampled, their mean ages ± standard deviations (SD) were 27 ± 9 years, and all subjects were found to be healthy. Of the women, 22 used oral contraceptives at lumbar puncture, 29 did not, whereas data were missing for three female participants. Except for oral contraceptives, all subjects were drug-free at lumbar puncture. Eight to twenty years after the first investigation, all subjects were re-interviewed to re-assess the psychiatric morbidity as previously described [20, 23]. At this interview, whole blood was drawn from all participants. At the second investigation, 43 of the subjects were found to have experienced various DSM-III-R psychiatric lifetime diagnoses. The study was conducted in accordance with the Declaration of Helsinki and approved by the Ethics Committee of the Karolinska University Hospital. Informed consent was obtained from all the participating subjects.

### CSF monoamine metabolite concentrations

CSF samples (12.5 ml) were obtained by lumbar puncture and analyzed as previously described [22, 45, 47]. Briefly, the samples were drawn between 8 and 9 a.m. with the subjects in the sitting or recumbent position, after at least 8 h of bed rest and absence of food intake or smoking. 5-HIAA, HVA and MHPG concentrations were measured by mass fragmentography with deuterium-labeled standards. Back-length was defined as the distance between the external occipital protuberance and the point of needle insertion.

### DNA analysis

Genomic DNA was extracted from whole blood [12]. Four *DAOA* SNPs (rs2391191 or M15, rs778294 or M19, rs3918342 or M23, rs1421292 or M24), previously reported to be associated with schizophrenia, were selected and genotyped at the SNP Technology Platform at Uppsala University and Uppsala University Hospital, Sweden (http://www.genotyping.se), using the Illumina BeadStation 500GX and the 1536-plex Illumina Golden Gate assay (Illumina Inc., San Diego, CA, USA) as previously described [21].

### Statistical analysis

Hardy–Weinberg (HW) equilibrium was tested using Fisher’s exact test as implemented in PEDSTATS [51]. Linkage disequilibrium (D′ and *r* ^2^) between SNP pairs was determined with Haploview 4.0 [3]. Allele association between *DAOA* SNPs and CSF monoamine metabolite concentrations was tested with a general linear model (Proc GLM, SAS/STAT^®^ software, version 9.1.3, SAS institute Inc., Cary, NC, USA), where concentration was modeled as a linear function of the allele count (of each SNP separately) and one or more covariates (single-marker association).

Covariates were selected by preliminary analysis excluding genetic markers. That is, the effect of potentially important confounders (back-length, weight, gender, age at lumbar puncture and presence of a lifetime psychiatric diagnosis) on CSF monoamine metabolite concentrations was evaluated by forward stepwise selection, as previously described [20]. Confounders that explained a significant part of systematic variation in CSF concentrations (*P* < 0.1) were included as covariates in the genetic association analysis. Thus, back-length and presence of a lifetime psychiatric diagnosis were used as covariates in the analysis of 5-HIAA and HVA concentrations, whereas back-length and gender were included in the analyses of MHPG. We tested the normal distribution of residuals with the Anderson–Darling test, and residuals were approximately normally distributed after square root (5-HIAA, HVA) and logarithmic (MHPG) transformations. Correction for multiple testing was performed through random permutation of the four marker genotypes among individuals and recalculation of the *P* values for the 12 tests for each permuted data set (1,000 permuted data sets). The corrected *P* value was then calculated as the fraction of permutated data sets where the minimum *P* value from the 12 tests was equal to, or smaller than, the observed *P* value. Moreover, rs3918342, showing the strongest association with HVA, was selected for further analysis, applying a dominant model of segregation.

## Results

**Fig. 2:**
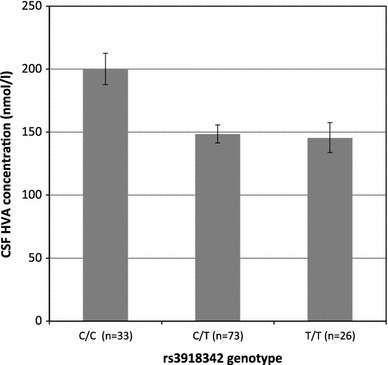
Cerebrospinal fluid (CSF) homovanillic acid (HVA) concentration in healthy subjects as a function of the number of rs3918342 T-alleles (corrected *P* value = 0.013). Least square means and standard errors are given

The mean (SD) concentrations of the three monoamine metabolites were: HVA, 170.2 (72.3) nmol/L; 5-HIAA, 91.7 (37.4) nmol/L; MHPG, 41.6 (8.2) nmol/L. Two of the investigated polymorphisms, rs3918342 (Fig. 2) and rs1421292, were found to be significantly associated with CSF HVA concentrations with corrected *P* values 0.013 and 0.043, respectively (Table 1). Rs3918342 was nominally associated with CSF 5-HIAA concentration, but this association was not statistically significant when accounting for the number of tests conducted. No polymorphisms were associated with MHPG concentrations.

**Table 1:** Allele association between d-amino acid oxidase activator (*DAOA*) single-nucleotide polymorphisms (SNPs) and 5-hydroxyindoleacetic acid (5-HIAA), homovanillic acid (HVA) and 3-methoxy-4-hydroxyphenylglycol (MHPG) concentrations in cerebrospinal fluid (CSF)

SNP	Base^a^	MAF^b^	HW^c^	Genotype count		5-HIAA			HVA			MHPG		
						Mean concentration (nmol/l)	AddVal	*P* value	Mean concentration (nmol/l)	AddVal	*P* value	Mean concentration (nmol/l)	AddVal	*P* value
Rs2391191	(G/A)	0.42	0.28	G/G	48	85	−0.02	0.83	170	−0.14	0.20	42	−0.14	0.24
				A/G	58	86			155			41		
				A/A	26	83			152			40		
Rs778 294	(C/T)	0.28	0.39	C/C	70	88	−0.11	0.40	161	−0.03	0.84	42	−0.20	0.13
				C/T	49	82			158			40		
				T/T	12	83			159			40		
Rs391 8342	(C/T)	0.47	0.29	C/C	33	103	−0.27	0.03	200	−0.40	0.001^d^	43	−0.06	0.68
				C/T	73	78			149			39		
				T/T	26	84			145			43		
Rs142 1292	(T/A)	0.44	0.38	T/T	39	96	−0.21	0.09	190	−0.35	0.004^d^	44	−0.17	0.20
				A/T	71	80			149			39		
				A/A	22	84			147			43		

For each monoamine metabolite, the mean CSF concentration per genotype is listed together with effect size (AddVal) due to the presence of one minor allele (given as standard deviations), and the corresponding *P* value from single-marker association analysis

^a^Major/minor allele

^b^Minor allele frequency

^c^Probability of deviation from Hardy–Weinberg equilibrium

^d^Associations that remains significant after correction for multiple testing

The two SNPs associated with HVA concentrations, rs3918342 and rs1421292, are in strong linkage disequilibrium (LD; *r* ^2^ = 0.80) in Caucasians (HapMap, release 24). In the Scandinavian population, the two markers are in almost complete LD (*r* ^2^ = 0.99) [21], and thus, they captured the same association signal in this study. Consequently, rs1421292 explained no additional variation in HVA concentration (*P* = 0.95) on top of that explained by rs3918342.

Carriers of the rs3918342 T allele (both C/T and T/T) had 50 nmol/l lower HVA mean concentrations compared with C homozygotes; no difference in HVA mean concentrations was found between C/T and T/T (Fig. 2). This pattern is consistent with a dominant model of segregation (T allele dominant), and as expected, this model resulted in a substantial decrease in the uncorrected *P* value for the association between rs3918342 and HVA (from 0.0016 to 0.0001).

## Discussion

In the present study, two *DAOA* polymorphisms, rs3918342 and rs1421292, were significantly associated with CSF HVA concentrations. Rs3918342 and rs1421292 are located 42 and 55 kbp from the 3′ end of *DAOA*, respectively, and are in strong linkage disequilibrium. Rs3918342 and rs1421292 have not been ascribed any functionality and were not found to be associated in strong LD (*r* ^2^ > 0.6) with any SNP within the *DAOA* borders. However, both were in strong LD with some intergenic SNPs within 500 kbp from rs3918342 (HapMap release 24). The associated intergenic SNPs lack currently known function or association with mental disorders.

During the past decades, a large number of CSF candidate markers, including the monoamine metabolite HVA, have been investigated with regard to their relevance to schizophrenia [48]. HVA concentrations have been reported to be significantly lower in drug-free schizophrenic patients compared with controls [6, 50]. Both quetiapine and olanzapine administrations have been associated with a significant increase in CSF HVA [33, 43], whereas haloperidol withdrawal resulted in a significant decrease in CSF HVA [5]. Thus, decreased HVA concentration appears to be related to schizophrenia.

There are several studies suggesting that a locus located near the 3′ end of *DAOA* is associated with phenotypes characteristic of schizophrenia or the progression of the disease. For example, both rs3918342 and rs1421292 have been associated with attention and memory impairments in schizophrenic individuals [14]. Rs3918342 has been associated with decreased hippocampal activation and increased prefrontal activation in subjects at high genetic risk of schizophrenia [15], as well as temporal lobe and amygdala gray matter reduction [53]. Furthermore, rs1421292 has been associated with brain activation in the right middle temporal gyrus and the right precuneus in healthy individuals [27]. Rs3918342 has been significantly associated with schizophrenia in independent studies [4, 7, 25]. However, meta-analysis of rs3918342 suggests that the association is restricted to populations of Caucasian origin and that the effect size is small (odds ratio = 1.03, non-significant; http://www.szgene.org) [2].

We found the TT genotype of rs3918342 to be strongly associated with decreased HVA concentrations, and we note that it is also this genotype that has been associated with attention and memory impairments in schizophrenic individuals [14], decreased hippocampal activation and increased prefrontal activation in subjects at high genetic risk of schizophrenia [15] as well as temporal lobe and amygdala gray matter reduction in bipolar patients [53]. Thus, it is possible that a disturbed dopamine turnover, reflected by decreased HVA levels, may be a mechanism behind one or several of the cognitive, neurological and brain morphological phenotypes previously associated with the rs3918342 TT genotype.

In conclusion, our results suggest that *DAOA* gene variation significantly affects dopamine turnover in CNS of healthy controls. Further research is needed in order to replicate our findings in healthy controls and, moreover, to find out whether the present associations can also be observed in schizophrenia and other psychiatric disorders.
